# Barriers to scaling up hepatitis C treatment in Malaysia: a qualitative study with key stakeholders

**DOI:** 10.1186/s12889-022-12786-w

**Published:** 2022-02-21

**Authors:** Huan-Keat Chan, Mohamed Azmi Hassali, Noor Syahireen Mohammed, Azlina Azlan, Muhammad Radzi Abu Hassan

**Affiliations:** 1 Discipline of Social and Administrative Pharmacy, School of Pharmaceutical Sciences, Universiti Sains Malaysia, Gelugor, Penang Malaysia; 2 Clinical Research Center, Sultanah Bahiyah Hospital, Alor Setar, 05460 Alor Setar, Kedah Malaysia; 3 Public Health Division, State Health Department, Alor Setar, Kedah Malaysia; 4 Medical Department, Sultanah Bahiyah Hospital, Alor Setar, Kedah Malaysia

**Keywords:** Antiviral agents, hepatitis C, Malaysia, Public health, Health services accessibility

## Abstract

**Background:**

While the availability of generic direct-acting antivirals (DAAs) opens the door for large-scale treatment, the care for people living with hepatitis C virus (HCV) in Malaysia is shifting toward a tripartite partnership between the public health system, correctional settings and civil society organizations (CSOs). This study aimed to explore the barriers to scaling up HCV treatment in Malaysia from the perspective of key stakeholders.

**Methods:**

Eighteen focus-group discussions (FGDs) were conducted with 180 individuals, who actively engaged in coordinating, executing or supporting the implementation of the national strategic plan for HCV. An analytical framework was adapted to guide the data collection and thematic analysis. It covered four key aspects of HCV treatment: geographical accessibility, availability, affordability and acceptability.

**Results:**

Movement restrictions in times of coronavirus disease 2019 (COVID-19) outbreaks and being marginalized translated into barriers to treatment access in people living with HCV. Barriers to treatment initiation in health and correctional settings included limited staffing and capacity; disruption in material supply; silos mentality and unintegrated systems; logistical challenges for laboratory tests; and insufficient knowledge of care providers. Although no-cost health services were in place, concerns over transportation costs and productivity loss also continued to suppress the treatment uptake. Limited disease awareness, along with the disease-related stigma, further lowered the treatment acceptability.

**Conclusions:**

This study disclosed a series of supply- and demand-side barriers to expanding the treatment coverage among people living with HCV in Malaysia. The findings call for strengthening inter-organizational collaborations to overcome the barriers.

## Background

Hepatitis C virus (HCV) infection remains a prominent global health concern. More than 70 million people are living with HCV, and nearly 400,000 people die of its chronic complications annually [1]. Malaysia has a moderate HCV burden, with an estimated anti-HCV prevalence of 1.9% [2]. The key risk factors of HCV in Malaysia include the history of injecting drugs and imprisonment [3]. In line with the goal of the World Health Organization (WHO), Malaysia is paving a path toward eliminating HCV as a public health threat by 2030 [4].

The public healthcare system in Malaysia, under the lead of the Ministry of Health, has a long history of providing no-cost health services through hospitals and primary healthcare (PHC) centers [5]. However, the use of costly direct-acting antivirals (DAAs) for HCV treatment was once restricted by the limited public health budget [6]. Following unfruitful price negotiations, Malaysia decided to invoke compulsory licensing to enable the import of generic sofosbuvir in late 2017 [7]. In line with the WHO’s recommendation, Malaysia subsequently introduced a DAA-based regimen consisting of sofosbuvir and daclatasvir to be used as the standard hepatitis C treatment in public hospitals [8, 9]. To further scale up HCV screening and treatment, Malaysia also launched a 5-year national strategic plan in 2019, which marked the transition of HCV care from a hospital-based model to a community-based model [10, 11].

**Fig. 1:**
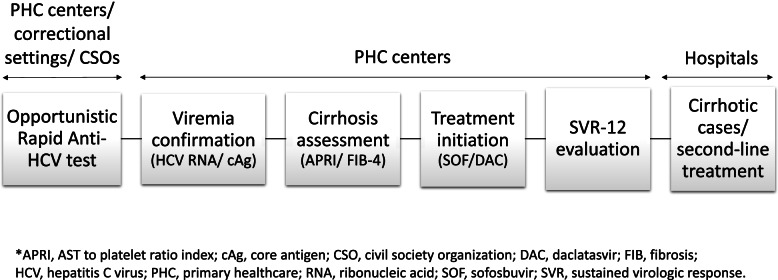
Current hepatitis C care model in Malaysia

Service decentralization and multi-stakeholder partnerships are the very essence of the new HCV care model in Malaysia (Fig. 1) [10]. Since 2019, the Ministry of Health has been working collaboratively with correctional settings and civil society organizations (CSOs) to provide HCV care. Rapid anti-HCV screening test is actively performed on high-risk individuals not only in PHC centers, prisons and drug rehabilitation centers but also through outreach programs of CSOs. The services offered by PHC centers widely range from viremia confirmation to the initiation of pharmacological treatment, while hospitals focus on treating referrals (cirrhosis and treatment failure cases) and providing laboratory supports (HCV viral load test and genotyping) for PHC centers.

Despite the expanded treatment coverage [9], the achievement of Malaysia is still far from meeting the global HCV elimination goal. The attrition across the cascade of HCV care also remains an unresolved issue. This study was designed as part of the interim review of the national strategic plan for HCV, aiming to explore the barriers to scaling up DAA-based treatment in Malaysia from the perspective of key stakeholders.

## Methods

### Study team

This was a qualitative study. It was reported in line with the Consolidated Criteria for Reporting Qualitative Research (COREQ) [12]. The study team comprised a gastroenterologist, a public health officer, two pharmacists and a university professor. They all had experience in conducting qualitative health research.

### Data collection and participants

**Fig. 2:**
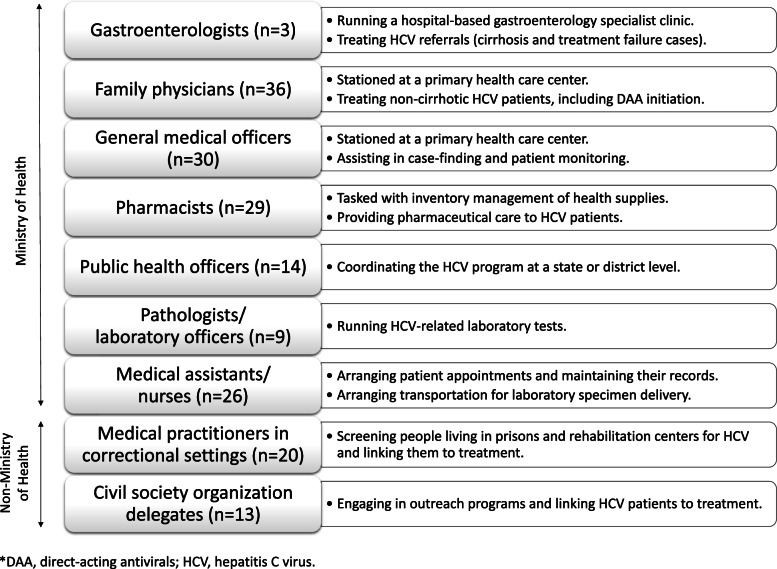
Eligible participants for the study

Eighteen focus-group discussions (FGDs) were performed between September 2020 and March 2021, with the aim to gather a rich blend of experience-based perspectives [13, 14]. The purposive sampling method [15] was used to identify participants from across the country, who were known to have an active engagement in coordinating, executing or supporting the implementation of the national strategic plan for HCV (Fig. 2). They were invited to a FGD held in one of the four selected locations in Peninsular Malaysia.

The semi-structured discussion guide (Table 1) used to facilitate the FGDs was designed based on an analytical framework [16], which was originally intended to address limited access to health services in low-income countries. This framework was selected for its coverage of four key aspects of HCV care, namely geographical accessibility, availability, affordability and acceptability. The FGDs were recommended to center on (i) HCV screening and outreach to key population, (ii) diagnosis and laboratory tests, (iii) supply sustainability and inventory management, and (iv) pharmacological treatment initiation and follow-up. The discussion guide underwent a pilot test with a multidisciplinary team of five members, after which a few questions were slightly modified and probes were identified.

**Table 1:** Semi-structured discussion guide

Scope of discussion	Questions and probes	Dimension of treatment access
Screening and outreach to key populations	Who are the key populations of HCV in your area, and what have been the challenges in linking them to care? *(Probes: distance, movement restriction)*	Geographical accessibility
	What have been the challenges in HCV screening and case-finding in your setting? *(Probes: patient load, staffing, facilities, system, knowledge, communication, culture)*	Availability/ acceptability
	What have been the challenges in referring individuals with a positive rapid screening test result to other settings for confirmatory testing and further management? *(Probes: logistical barriers, patient load, staffing, facilities, system, communication, culture)*	Geographical accessibility availability/ acceptability
Diagnosis and laboratory tests	What have been the challenges in HCV diagnosis and laboratory tests in your setting? (*Probes: patient load, staffing, facilities, system*)	Availability
	What have been the challenges in communicating the test results with other settings? *(Probes: system, turnaround time, communication, culture)*	Availability/ acceptability
Supply sustainability and inventory management	Are there any issues with the supply of direct-acting antivirals, rapid screening test kits and laboratory test reagents in your setting? (*Probes: cost, budget, amounts*)	Availability/ affordability
Pharmacological treatment and follow-up	What have been the challenges in initiating HCV treatment and evaluating its outcomes in your setting? (*Probes: awareness, attitude, refusal, system, skills*)	Availability/ acceptability
	Although the treatment is provided free of charge, do you think there are any other indirect costs discouraging people living with HCV from receiving treatment? *(Probes: multiple visits, transportation)*	Affordability
*HCV* hepatitis C virus		

The FGDs were performed in a conference room. Each FGD took 90 to 120 min, and was participated by eight to 12 individuals from different professional backgrounds. The authors assisted in the FGDs as facilitators (MRAH and AA) and note-takers (HKC and NSM). Each participant only took part in one FGD. All the FGDs were conducted in the Malay language and audio-recorded. They were immediately transcribed verbatim after each session, and the transcripts were returned to two participants who were familiar with the subject matter to check for accuracy.

### Data analysis

A Microsoft Excel spreadsheet was used to manage the qualitative data. The principles of deductive thematic analysis, constant comparison and investigator triangulation were applied for the data analysis [17–20]. Two authors (HKC and NSM) independently generated codes from the transcripts and fitted them into the thematic structure of the analytical framework [16]. They also independently studied the links between the codes and developed the thematic map. Any disagreements in the proposed themes and subthemes were then resolved by consensus. For the purpose of publication, the quotes selected to exemplify the themes were also translated into English by the same two authors. Data saturation was not pursued in this study given the aim to gather the broadest perspective possible from the participants.

## Results

Eighteen FGDs (FGD 1-18) were conducted. The 180 participants represented all the targeted key stakeholders (Fig. 2). The FGDs yielded four themes (Table 2), which covered both supply- and demand-side barriers to scaling up DAA-based treatment in Malaysia.

**Table 2:** Summary on themes and subthemes emerging from the focus group discussions

Themes	Subthemes	Summary
1. Limited access to health facilities	(a) Movement restrictions during COVID-19 outbreaks (b) Marginalized key populations	Major factors limiting the access of people living with HCV to primary healthcare clinics and hospitals.
2. Gaps in HCV treatment delivery	(a) Limited staffing and capacity (b) Disruption in material supply (c) Silos mentality and unintegrated systems (d) Logistical challenges for laboratory tests (e) Insufficient knowledge of care providers	Institutional insufficiencies affecting the delivery of HCV care.
3. Free yet unaffordable treatment	(a) Transportation costs (b) Productivity loss	Reasons of life pressure among people living with HCV suppressing the uptake of no-cost treatment.
4. Suboptimal acceptability of treatment	(a) Limited disease awareness and treatment adherence (b) Disease-related stigma	Causes of pharmacological treatment not widely accepted in people living with HCV.
*COVID-19* coronavirus disease-2019, *HCV* hepatitis C virus		

### Theme 1: Limited access to health facilities

#### Movement restrictions during coronavirus disease 2019 (COVID-19) outbreaks

Malaysia had been hard-hit by the COVID-19 pandemic for almost a year at the point of the study. Intermittent travel restrictions, or formally known as movement control orders, were consistently cited as the “*key reason of HCV patients not seeking treatment and defaulting on their clinic appointments*” (family physician, FGD 5). After observing a series of lockdowns, a gastroenterologist (FGD 6) also highlighted that *“the condition was even more challenging to those who were tested positive for anti-HCV elsewhere (in correctional settings or by CSOs) and needed to travel far to health settings for treatment*”. The “*prohibition of vehicle sharing”* (medical assistant, FGD 12), coupled with the *“fear of COVID-19*” (general medical officer, FGD 4), further discouraged people living with HCV from care seeking. As simplifying HCV care became more relevant than ever, a family physician (FGD 4) perceived that *“a lot more efforts could be put into integrating HCV testing and treatment with other health services”* in response to the ongoing pandemic.

One of the key populations of HCV in Malaysia was people who inject drugs (PWID), who were described to *“have a Tom-and-Jerry-like relationship with police”* (public health officer, FGD 13). The set-up of more police roadblocks during the travel restrictions was also linked to their reluctance to travel. A pharmacist (FGD 13) shared her observation that *“a few patients did not turn up for prescription refills simply because of their fear of getting arrested for drug possession at the police roadblocks”*. Although the volatility of COVID-19 pandemic impeded the provision of HCV care for PWID, a mechanism to *“allow information exchange between health settings and enforcers of the movement control orders”* (FGD 8, pharmacist) was yet to be established.

#### Marginalized key populations

Methadone replacement therapy for PWID had been provided in PHC centers since 2005. As active case-finding for HCV was also mainly performed in PHC centers, it was perceived that the availability of DAAs “*disproportionately benefited the clients of the methadone replacement therapy program*” (gastroenterologist, FGD 13). Thus, the participants expressed their concerns over the inadequate attention devoted to other hard-to-reach key populations of HCV, who *“did not seek treatment when HCV showed no symptoms”* (general medical officer, FGD 6) and *“were not easily contactable even if they did”* (public health officer, FGD 6).

A family physician (FGD 8) observed that the marginalized key populations of HCV in Malaysia included “*people with a history of living in prisons and drug rehabilitation centers; PWID hanging around ‘injection ports’ (the places where PWID traded and injected drugs); sex workers; people with tattoos; the transgender population; men having sex with men; and fishermen”*. Within this context, a public health officer (FGD 10) underlined *“the lack of outreach programs to perform on-site HCV testing on these populations”.* Furthermore, another public health officer (FGD 2) described CSOs as *“the window into neglected key populations of HCV”* and expected them to *“play a more active role in linking these populations to care”*.

### Theme 2: Gaps in HCV treatment delivery

#### Limited staffing and capacity

Public health settings generally did not face a workforce shortage, especially after “*more and more general medical officers were stationed in PHC centers and hospitals*” (family physician, FGD 3). In contrast, health services in prisons and drug rehabilitation centers were run by small medical teams appointed by the Ministry of Home Affairs. Understaffing and multitasking were cited as the major challenges in performing HCV screening and linking those with a positive test result to care in correctional settings. A medical practitioners (FGD 7) shared his experience as follows: *“I served in a prison with more than 3,000 people, but there was only a small medical team of three doctors and three assistants. We were obliged to manage a wide range of complicated illnesses, including mental illnesses”*. A family physician (FGD 3) commented that resource sharing between ministries, such as by *“sending visiting physicians from PHC centers to assist prisons and drug rehabilitation centers”*, would have alleviated the problem. However, she added that such an attempt was often *“limited by a strict protocol to receive external care providers”* in correctional settings.

#### Disruption in material supply

The DAAs and rapid screening test kits used in both health and correctional settings were centrally acquired by the Ministry of Health at the point of the study. Despite the reassurance that *“the budget allocated for HCV treatment had been sufficient since generic DAAs were brought in”* (pharmacist, FGD 10), the concern over *“occasional disruption in the supply of DAAs and rapid screening test kits”* (general medical officer, FGD 16) was raised. A family physician (FGD 16) pointed out that “*patients had to be placed on the waiting list when PHC centers ran out of medications”*. A medical practitioner from a prison (FGD 7) also admitted that his team *“had no choice but withheld the HCV screening”* when the supply of rapid screening test kits was disrupted. Nevertheless, a pharmacist (FGD 5), who was tasked with the procurement of health supplies for a few PHC centers, believed that the aforementioned challenges were *“manageable”* and could be addressed by *“improving the inventory management of both DAAs and rapid screening test kits”*.

#### Silos mentality and unintegrated systems

The absence of an effective mechanism to facilitate the inter-organizational communication was also highlighted. Although it was believed that the public health system in Malaysia “*has everything set”* (gastroenterologist, FGD 1), a family physician (FGD 8) opined that the country “*still lacked a systematic approach to track patients moving from one place to another to seek care”*. A medical officer (FGD 12) added that *“neither a joint committee nor at least a social media chat group to coordinate HCV care activities was available”*. Such limitations occasionally translated into repetition of screening and laboratory tests, treatment interruption, missed opportunities to initiate pharmacological treatment and even medical errors. Another family physician (FGD 11) echoed the view and gave an example of a patient newly released from a prison, who “*did not have any document showing that he was receiving HCV treatment, and was therefore put on a new course of DAAs unintentionally*”. A pharmacist (FGD 15) also shared his observation that *“treatment was commonly discontinued when patients were under arrest, detention or imprisonment”*, especially when the officers in correctional settings were uninformed of their health conditions.

#### Logistical challenges for laboratory tests

While viremia confirmation and HCV genotyping were both performed in hospital labs, the delay in treatment initiation in PHC centers was partly attributed to the lengthy laboratory turnaround time and logistical hassles associated with the specimen delivery. According to a family physician (FGD 6), *“the turnaround time was at least two weeks for the HCV RNA (ribonucleic acid) test, and a few months for the HCV genotyping”.* Sometimes it could take even longer when *“the hospitals were overstretched with a high patient volume”* (laboratory officer, FGD 6), or simply because *“vehicles used for specimen delivery broke down”* (medical assistant, FGD 15). Aside from the lengthy turnaround time, arranging transportation for specimen delivery was also pictured by a nurse (FGD 12) as *“a disruptive and tedious process”*, which could be frustrating and suppress treatment initiation. As it was unlikely to substantially increase the laboratory capacity of PHC centers within a short time, simple and yet practical approaches, such as *“communicating the results immediately after laboratory tests by phone or email”* (laboratory officer, FGD 16) and *“sharing vehicles for specimen delivery”* (medical assistant, FGD 3), could be adopted to improve the laboratory efficiency.

#### Insufficient knowledge of care providers

While a major part of the responsibility for HCV care was placed on PHC centers, the participants highlighted the lack of knowledge and confidence in managing the disease among less experienced care providers. A general medical officer (FGD 9) shared his observation that some of his peers, who were expected to screen high-risk individuals and refer them to family physicians for treatment initiation, were *“unfamiliar with HCV and its risk factors”*. It was also disclosed that inappropriate management of HCV, particularly in the forms of *“collecting blood for unjustified laboratory tests”* (family physician, FGD 9) and “*referring uncomplicated, non-cirrhotic cases to hospitals*” (general medical officer, FGD 10), still occasionally occurred. At the same time, HCV, along with DAAs, was also deemed to be *“a new thing to pharmacists”* (FGD 15, pharmacist). One of them (FGD 12), who ran a medication therapy adherence clinic for HCV, observed that most pharmacists stationed in PHC centers were *“not equipped with sufficient knowledge to provide patients with effective counseling”*. Hence, *“more guidance and training”* (general medical officer, FGD 9) for care providers were required.

### Theme 3: Free yet unaffordable treatment

#### Transportation costs

The existing HCV care model required multiple visits to PHC centers for screening test, viremia confirmation, cirrhosis status assessment, treatment initiation, prescription refills and monthly follow-up. Even though the treatment was provided free of charge, a CSO delegate (FGD 9) cited the need to regularly locate fund for transportation *“as a common source of stress and reason of refusing treatment”*. A family physician (FGD 10) elaborated on this matter as follows: *“Many of people living with HCV were from disadvantaged backgrounds. They either did not have a stable job or were unemployed. Paying even as little as 5 ringgits (the minimum one-way fare) for Grab (ride-hailing services) could be extremely burdensome for them”*. He also wished that CSOs could *“expand their programs to cover the underprivileged members of the community and bring them to health settings for treatment”.*

#### Productivity loss

As most people living with HCV were in the productive phase of their lives, the need of making multiple visits to health settings also cost them and their family considerable economic opportunities. A family physician (FGD 9) shared a case of a newly employed factory worker under HCV treatment, who was *“questioned by his supervisor and almost sacked from his job”* for frequent absenteeism. At the same time, the husband of another patient *“was unable to work (as a taxi driver) when sending her to the hospital for monthly follow-up visits”* (medical assistant, FGD 7). Therefore, a public health officer (FGD 4) stressed the need *“to minimize the number of scheduled visits so that the patients could go to work”*, such as through the use of telemedicine and mail pharmacy services for prescription refills.

### Theme 4: Suboptimal acceptability of treatment

#### Limited disease awareness and treatment adherence

Although DAAs were claimed to be *“very effective”* (FGD 15, pharmacist) and *“highly appreciated”* (family physician, FGD 16), a public health officer (FGD 3) found that people living with HCV still generally *“knew very little about the disease, let alone the fact that it was curable”*. A general medical officer (FGD 10) also admitted that he “*had a hard time convincing some of them to receive a screening test*”. Moreover, it was emphasized that *“the consequences of non-adherence to treatment must be reiterated”* (pharmacist, FGD 12), especially to those who were found to *“misplace their medications”* (pharmacist, FGD 12), *“skip doses”* (pharmacist, FGD 15), *“turn up late for follow-up”* (family physician, FGD 10), and *“continue substance use”* (FGD 15, CSO delegate).

#### Disease-related stigma

The suboptimal uptake of HCV screening and treatment was also partly attributed to the stigmatization of the disease. *“The public often related HCV to drug abuse”* (CSO delegate, FGD 8), *and “some patients had been hesitant about receiving treatment to avoid discrimination”* (family physician, FGD 16). Apart from that, “*the lack of sensitivity to the feeling of patients”* (family physician, FGD 5) and *“the tendency to be judgmental about how they contracted HCV”* (family physician, FGD 16) in some less experienced care providers might have unintentionally triggered treatment rejection.

## Discussion

The uncertainty of the progress of Malaysia toward eliminating HCV as a public health threat has received considerable attention, particularly after realizing the need to battle the disease while dealing with the ongoing COVID-19 pandemic [21]. This study represents the first attempt of Malaysia to revisit the national response to HCV and characterize the challenges to expanding the treatment coverage. As issues related to HCV care have been explored mainly from the patient perspective [22–26], this study also adds to the existing literature by addressing both supply- and demand-side barriers in the context of a tripartite partnership between the public health system, the criminal justice system and CSOs.

Malaysia, along with many countries dealing with competing priorities and resource constraints, is looking for opportunities to scaling up the use of DAAs. Although exorbitant drug costs used to pose a substantial financial challenge to Malaysia [27, 28], budgetary pressure was not cited as a barrier to scaling up HCV treatment in this study. It was reaffirmed by the study participants that sufficient financial support for HCV care had been received from the government over the years. Such finding implies the success of Malaysia in its drug price control policy, which was achieved mainly by the application of compulsory licensing on a patented DAA [9]. Nonetheless, a lesson worth learning from this study is that lowering the prices of DAAs alone would not assure the massive expansion of treatment coverage.

Some barriers to scaling up HCV treatment uncovered in this study overlapped with those listed by Mendizabal et al. [29], including the limited accessibility of healthcare and stigmatization of the disease. However, this study also draws an equal attention to the gaps in the delivery of HCV care. It is noteworthy that most challenges associated with the health settings are likely to be “low hanging fruit”, as they lie completely within the capacity of the Ministry of Health and are manageable by resource optimization. The knowledge of care providers, for instance, could be improved through proper guidance and training. Furthermore, the laboratory efficiency could be easily enhanced by sharing vehicles for specimen delivery and instant communication of test results. While Malaysia is pinning its hopes on PHC centers to battle HCV, it is also essential to establish a mechanism for information exchange between them.

This study also provides insight into the translation of real-life struggles among people living with HCV into bottlenecks in case-finding and treatment initiation. It was reported that the need of making multiple visits to health settings, coupled with the resultant financial burden, had either prompted the loss of patients during the care process or distanced them from care seeking. The findings are similar to those of a study on people living with human immunodeficiency virus, suggesting a strong link between social vulnerabilities and the low adherence to treatment [30]. Moreover, this study demonstrated that the impact of the COVID-19 pandemic on the provision of HCV care was as significant in Malaysia as in the rest of the world [31]. As such a challenge is unprecedented and likely to last long, the initiatives to simplify the treatment procedure and reduce the number of scheduled visits, as foregrounded by the study participants, are timely and relevant.

The high burden of viral hepatitis in correctional settings due to the repressive drug laws is not a new issue in Malaysia. Given that most PWID in Malaysia had a history of staying in either prisons or drug rehabilitation centers in their lifetime [32], it is reasonable for the Ministry of Health to continuously support the HCV care activities in these settings with health supplies. Consistent with the viewpoint of Culbert et al. [32], the study participants also attributed unaddressed health needs in people living in correctional settings partly to the workforce shortage. As much as the visiting family physicians from PHC centers could be helpful, it is important to streamline the protocol of accepting external care providers in correctional settings. Another potential strategy to accelerate the initiation of HCV treatment among people living in correctional settings without increasing the demand for labor is by performing the screening at the point of their admission [33].

In addition to influencing policies and advocating drug accessibility [34], this study calls on CSOs to play a more active role in HCV care. As in other countries [35, 36], disruption in continuum of HCV care among people living in correctional settings is common in Malaysia. CSOs could fill the gap by linking them up with PHC centers after they are released. To approach hard-to-reach populations who would otherwise not seek care, the Ministry of Health could also capitalize on the outreach programs of CSOs to identify people in need of HCV treatment. Such programs were also shown to be useful to improve their retention in care, especially when the HCV screening services and treatment were offered concurrently with harm reduction activities and education [37].

This study was subject to a few limitations. The FGDs were staged in Peninsular Malaysia, and no representatives from East Malaysia were invited due to travel restrictions in times of the COVID-19 pandemic. Therefore, further investigation to give a full picture of barriers to scaling up HCV treatment in all regions across the country is warranted. Additionally, the study participants were mainly those who were experienced in providing HCV care. Hence, the findings might not capture the challenges in clinical and correctional settings, which had not actively provided HCV treatment at the point of the study.

## Conclusions

This study locates the areas requiring attention in order to eliminate HCV as a public health threat in Malaysia. After political buy-in brought an early success in enabling the access to DAAs at a tremendously low cost, the study findings point to the need to further expand the treatment coverage, mainly by strengthening collaborations between the public health system, correctional settings and CSOs.

## Data Availability

The datasets generated and analyzed during the current study are not publicly available due to privacy concerns but are available from the corresponding author on reasonable request.

## Abbreviations

COREQ: Consolidated Criteria for Reporting Qualitative Research
COVID-19: Coronavirus disease 2019
CSO: Civil society organization
DAA: Direct-acting antiviral
DOT: Directly observed therapy
FGD: Focus group discussion
HCV: Hepatitis C virus
PHC: Public healthcare
PWID: People who inject drugs
RNA: Ribonucleic acid
WHO: World Health Organization
